# Nested Russian Doll-Like Genetic Mobility Drives Rapid Dissemination of the Carbapenem Resistance Gene *bla*_KPC_

**DOI:** 10.1128/AAC.00464-16

**Published:** 2016-05-23

**Authors:** Anna E. Sheppard, Nicole Stoesser, Daniel J. Wilson, Robert Sebra, Andrew Kasarskis, Luke W. Anson, Adam Giess, Louise J. Pankhurst, Alison Vaughan, Christopher J. Grim, Heather L. Cox, Anthony J. Yeh, the Modernising Medical Microbiology (MMM) Informatics Group, Costi D. Sifri, A. Sarah Walker, Tim E. Peto, Derrick W. Crook, Amy J. Mathers

**Affiliations:** a Modernizing Medical Microbiology Consortium, Nuffield Department of Clinical Medicine, John Radcliffe Hospital, Oxford University, Oxford, United Kingdom; b Icahn Institute and Department of Genetics and Genomic Sciences, Icahn School of Medicine, Mount Sinai, New York, New York, USA; c Food and Drug Administration, Laurel, Maryland, USA; d Division of Infectious Diseases and International Health, Department of Medicine, University of Virginia Health System, Charlottesville, Virginia, USA; e Office of Hospital Epidemiology, University of Virginia Health System, Charlottesville, Virginia, USA; f Public Health England, Microbiology Services, London, United Kingdom; g Clinical Microbiology, Department of Pathology, University of Virginia Health System, Charlottesville, Virginia, USA

## Abstract

The recent widespread emergence of carbapenem resistance in Enterobacteriaceae is a major public health concern, as carbapenems are a therapy of last resort against this family of common bacterial pathogens. Resistance genes can mobilize via various mechanisms, including conjugation and transposition; however, the importance of this mobility in short-term evolution, such as within nosocomial outbreaks, is unknown. Using a combination of short- and long-read whole-genome sequencing of 281 *bla*_KPC_-positive Enterobacteriaceae isolates from a single hospital over 5 years, we demonstrate rapid dissemination of this carbapenem resistance gene to multiple species, strains, and plasmids. Mobility of *bla*_KPC_ occurs at multiple nested genetic levels, with transmission of *bla*_KPC_ strains between individuals, frequent transfer of *bla*_KPC_ plasmids between strains/species, and frequent transposition of *bla*_KPC_ transposon Tn*4401* between plasmids. We also identify a common insertion site for Tn*4401* within various Tn*2*-like elements, suggesting that homologous recombination between Tn*2*-like elements has enhanced the spread of Tn*4401* between different plasmid vectors. Furthermore, while short-read sequencing has known limitations for plasmid assembly, various studies have attempted to overcome this by the use of reference-based methods. We also demonstrate that, as a consequence of the genetic mobility observed in this study, plasmid structures can be extremely dynamic, and therefore these reference-based methods, as well as traditional partial typing methods, can produce very misleading conclusions. Overall, our findings demonstrate that nonclonal resistance gene dissemination can be extremely rapid, presenting significant challenges for public health surveillance and achieving effective control of antibiotic resistance.

## INTRODUCTION

Although antibiotic resistance genes have been identified in ancient bacterial DNA (1), much of the recent, alarming increase in pathogen antimicrobial resistance is attributable to the dissemination of resistance genes via horizontal gene transfer (HGT) in response to selection imposed by widespread antibiotic use in medicine and agriculture (2, 3). Many resistance genes are located on plasmids that can be transferred between different bacterial strains or species, thus facilitating HGT (4). Furthermore, resistance gene mobility can be enhanced by integration into transposable elements, which are short stretches of DNA (several kilobases) that can autonomously mobilize between different genomic locations (5). However, the importance of HGT in short-term evolution is unclear, as capturing the processes in real time is challenging and outbreaks in health care settings are often thought to be dominated by clonal transmission (6–9).

Carbapenem resistance in Enterobacteriaceae has been recognized as a key threat to modern medicine (10, 11), as carbapenems often represent the therapy of last resort for serious infections (12, 13). One of the most prevalent carbapenem resistance genes is the Klebsiella pneumoniae carbapenemase (KPC) gene, *bla*_KPC_, first identified in 1996 and now endemic to many regions of the world (14). KPC is a beta-lactamase capable of hydrolyzing all beta-lactams, including penicillins, monobactams, cephalosporins, and carbapenems (15), leaving few treatment options for infected vulnerable hospitalized patients and resulting in worse treatment outcomes (16).

Most reports of *bla*_KPC_ involve K. pneumoniae multilocus sequence type 258 (ST258) (9, 17), which has been found globally, indicating that clonal dissemination of this resistant lineage has been an important factor in the spread of *bla*_KPC_ (9, 17–20). Nevertheless, *bla*_KPC_ has also been observed in other K. pneumoniae lineages, as well as other species of Enterobacteriaceae, suggesting that *bla*_KPC_ HGT has also played a role in resistance dissemination (21–25). As *bla*_KPC_ is often found on conjugative plasmids, some of which have been identified in multiple strains or species, this provides a likely mechanism for HGT (21, 26, 27). In addition, *bla*_KPC_ is usually present as part of the 10-kb Tn*3*-based mobile transposon Tn*4401*, which has been identified in various different plasmids, implicating Tn*4401* transposition as another mechanism contributing to *bla*_KPC_ spread (28, 29).

While Tn*4401* transposition and plasmid conjugation have been measured in the laboratory (28, 30, 31), the frequencies with which these processes occur within real-world ecosystems are not fully understood. In clinical contexts, it is often assumed that short-term evolution is dominated by clonal propagation, such that transmission chains generally involve a single pathogenic strain. However, if HGT is frequent relative to transmission (e.g., a “plasmid outbreak”), then linked patients may show variation in strain composition. If transposition is also frequent, then both the host strain and the resistance plasmid may show high variability within a single outbreak. As current surveillance strategies tend to focus on the host strain, it is important to establish the relevance of *bla*_KPC_ mobility within outbreak settings.

Traditional approaches to plasmid investigation, such as PCR-based replicon typing, are limited in resolution. Next-generation sequencing has been successfully applied to molecular epidemiological investigation of a number of pathogens at the host strain level; however, the application and limitations of this technology for transmission chains involving HGT are relatively unexplored. Whole-genome sequencing using short-read technologies (e.g., Illumina) has become cheap and accessible but is not ideal for plasmid analysis because of *de novo* assembly limitations, as it is often not possible to accurately reconstruct the genomic context surrounding repeated sequences (21, 32). Long-read sequencing (e.g., PacBio) can largely overcome this, often providing single-contig plasmid assemblies, but it is prohibitively expensive for many applications. Several studies have utilized reference-based methods for plasmid assembly or inference of plasmid structures using short-read data (33, 34); however, these approaches make the implicit assumption that plasmid structures are relatively stable. It will be important to understand the potential shortcomings of these assumptions in relation to mobile genetic elements, which may frequently be involved in plasmid rearrangements. Understanding when and how to successfully apply short- and/or long-read sequencing technologies to molecular epidemiology tracking will be important to the field as the incidence of HGT is increasingly recognized (35).

At our institution, *bla*_KPC_ was first identified in 2007 in a patient simultaneously colonized with *bla*_KPC_-positive K. pneumoniae and Klebsiella oxytoca harboring *bla*_KPC_ plasmids pKPC_UVA01 and pKPC_UVA02, respectively (36, 37). Since then, we have prospectively screened extended-spectrum cephalosporin-resistant/carbapenem-nonsusceptible isolates of all Enterobacteriaceae species for *bla*_KPC_, despite national guidelines that recommend that screening focus on carbapenem-nonsusceptible Klebsiella species and Escherichia coli (38–41). Here we describe the genetic basis of nonclonal *bla*_KPC_ emergence in a single hospital setting by using a combination of short- and long-read whole-genome sequencing methods to provide genomic characterization of 281 Enterobacteriaceae isolates from the first 5 years of this multispecies *bla*_KPC_ outbreak.

## MATERIALS AND METHODS

### Isolate collection and Illumina sequencing.

Isolates were prospectively collected from August 2007 to December 2012 through the Clinical Microbiology Laboratory of the University of Virginia Health System, which serves a 619-bed tertiary care hospital, outpatient clinics in central Virginia, and since August 2010, a 40-bed long-term acute care hospital. From April 2009, weekly surveillance by perirectal swab was performed in all inpatient units with historically high transmission or where there was a patient who was known to be colonized or infected with carbapenemase-producing Enterobacteriaceae (CPE) by previously described methods (40, 42, 43). Enterobacteriaceae isolates from nonsurveillance clinical samples that were flagged as possible extended-spectrum β-lactamase (ESBL) producing or had an ertapenem MIC of ≥1 μg/ml by automated susceptibility profiling (VITEK2; bioMérieux, Durham, NC) underwent carbapenemase phenotypic testing by the modified Hodge test (August 2007 to June 2008) or the indirect carbapenemase test (July 2008 to December 2012). Isolates with a positive carbapenemase phenotypic test and/or a meropenem or imipenem MIC of ≥1 μg/ml underwent *bla*_KPC_ PCR analysis as previously described (39).

A subset of 37 K. pneumoniae isolates, with corresponding sequence data, have been previously described (37). For the rest of the study isolates, Illumina sequencing, *de novo* assembly, mapping, and variant calling were performed as previously described (37), with some exceptions (see the supplemental material), and including the use of additional, species-specific references for mapping (see Table S5 in the supplemental material). A total of 281 isolates from 182 patients were available for analysis; for the exclusion criteria used for additional isolates, see the supplemental material.

### Classification to the species level.

Classification to the species level was performed by microbiological and sequenced-based methods (see the supplemental material for details).

### Phylogenetic analysis and strain classification.

There were 52 patients with multiple isolates of the same species. One of these (patient FK) carried two strains of K. pneumoniae that were highly divergent from each other (>20,000 chromosomal single-nucleotide variants [SNVs]), clearly representing a separate acquisition of *bla*_KPC_ by each strain. Excluding this divergent strain pair, the remaining cases had differences ranging from 0 to 60 SNVs (median, 2 SNVs). As these could plausibly represent clonal evolution within the patient, we conservatively chose to include only a single representative (the earliest isolate) for phylogenetic reconstruction, in order to avoid artificially inflating genetic clusters because of repeated patient sampling. Phylogenetic analysis was then performed separately for each species using PhyML (44) (see the supplemental material). Chromosomally distinct strains were defined by partitioning each phylogeny with a cutoff of ∼500 SNVs (see the supplemental material). On the basis of the molecular clock of Enterobacteriaceae (1 to 20 SNVs/chromosome/year) (6, 37, 45), we can be relatively confident that isolates belonging to distinct strains will not have a shared ancestor within the time frame of *bla*_KPC_ dispersal, and the number of distinct strains thus provides a conservative estimate of the number of distinct *bla*_KPC_ acquisition events.

### Long-read PacBio sequencing.

For long-read sequencing, 17 isolates were randomly chosen from the entire set of sequenced isolates (i.e., including patient duplicates). Long-read sequencing and initial *de novo* assembly were performed as previously described (37). Refinement of assemblies and closure of plasmid/chromosomal sequences was performed as described in the supplemental material.

**FIG 1:**
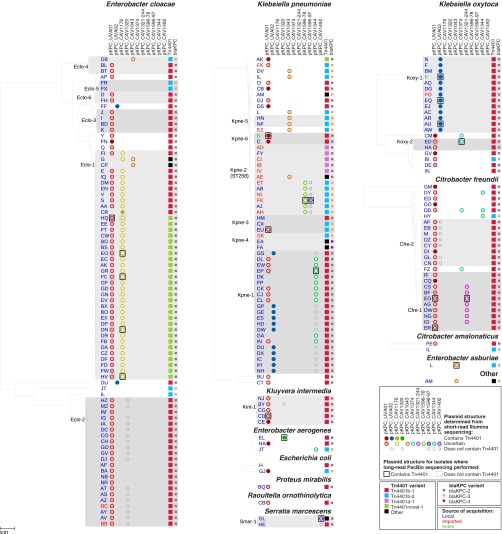
Diversity of bacterial species, strains, plasmids, and Tn*4401* variants. For each species, a phylogeny was generated from mapping to a species-specific chromosomal reference, after the deduplication of closely related isolates from the same patient (see Materials and Methods). Distinct strains are defined by a cutoff of ∼500 SNVs (see Materials and Methods); strains found in more than one patient are shaded gray. Circles show plasmid “presence” as determined from Illumina data, with the fill color indicating uncertainty about whether the plasmid contains *bla*_KPC_. Boxes show plasmid structures determined from long-read PacBio sequencing of 17 randomly chosen isolates, as well as the previously sequenced isolates from index patient B (37). Where the PacBio-sequenced isolate was excluded from the phylogeny as a patient duplicate, the plasmid structure of the corresponding closely related isolate from the same patient is shown. Tn*4401* and *bla*_KPC_ variants (Table 2) are indicated by large and small squares, respectively. The likely sources of *bla*_KPC_ acquisition, as determined from epidemiological data, are indicated by text color.

Since the isolates used for PacBio sequencing were randomly chosen from the set of all Illumina-sequenced isolates, some of them represented within-patient strain duplicates (see the previous section on phylogenetic analysis) and were therefore not included in the phylogenetic reconstruction. For display purposes (Fig. 1), the *bla*_KPC_ structure(s) determined by long-read PacBio sequencing for each of these isolates is shown alongside the representative isolate of the same strain from the same patient. In all of the cases, the representative isolate has the same short-read plasmid profile and Tn*4401* variant as the PacBio-sequenced isolate.

### Plasmid presence/absence classification.

The index *bla*_KPC_ plasmids pKPC_UVA01 and pKPC_UVA02, together with the additional nine distinct *bla*_KPC_ plasmids identified by long-read PacBio sequencing, were used as references to determine plasmid presence profiles for each isolate on the basis of the Illumina data. Plasmid presence was defined as ≥99% sequence identity over ≥80% of the length of the reference sequence, as determined by BLASTn comparison of each isolate's *de novo* assembly with the reference plasmid. The high identity cutoff was chosen to reduce false positives from sequences that are only distantly related (and therefore unlikely to have a common ancestor within the time frame of the outbreak), while the more permissive length cutoff allows for some rearrangement. It should be noted that the method does not take structural continuity into account.

### Analysis of Tn*4401* flanking sequences.

Where a plasmid was classified as being present in a particular isolate, it was not always certain to contain Tn*4401*. The plasmid presence classification was further refined as “containing Tn*4401*” if the isolate's *de novo* assembly supported Tn*4401* being present within the expected sequence context of that plasmid, “not containing Tn*4401*” if the plasmid was assembled without Tn*4401*, or “uncertain” if structure could not be determined from the *de novo* assembly. The identification of novel Tn*4401* insertion sites was also based on the *de novo* assemblies. These methods are described in detail in the supplemental material.

### Variation in Tn*4401*.

Tn*4401* isoform classification was performed by comparing each isolate's *de novo* assembly with the previously described isoform b reference sequence from EU176013.1 (29) using BLASTn to identify structural variation. SNVs were determined by mapping to a reference consisting of pKPC_UVA01 plus a species-specific chromosome as described above, followed by extraction of the Tn*4401* region. Variation is reported for all sites where at least one isolate had a nonreference call, including any ambiguity at that site in other isolates. Ambiguity at nonvariable sites is not reported, which may result in an underestimate of true variation. However, any resulting underestimation is likely to be very minor, as the proportion of called sites, excluding deleted regions described above, was >96% for all isolates.

### Epidemiological classification.

For epidemiologic analysis, patients were assigned a one- or two-letter code for deidentification. Routine perirectal surveillance cultures for silent colonization began in April 2009 (38, 40). Cases were classified as “imported” if they did not have any prior admission to the University of Virginia Medical Center/Long-term Acute Care Hospital (UVaMC) and either had a *bla*_KPC_-positive Enterobacteriaceae isolated within 48 h of admission or had a carbapenem-resistant Enterobacteriaceae culture before transfer to UVaMC with a subsequent isolate at UVaMC confirmed as *bla*_KPC_ PCR positive. The index case was also classified as imported. In the remaining cases, the source of *bla*_KPC_ acquisition was classified as “local.” The 48-h cutoff is arbitrary and may result in some misclassification if patients either acquire *bla*_KPC_ within the first 48 h of admission or if *bla*_KPC_ carriage/infection remains undetected for >48 h; however, this is expected to be minimal (see the supplemental material). Charts and patient contacts were reviewed by using bed tracing data and the electronic medical record. This study was approved by the University of Virginia Institutional Review Board (protocol 13558).

### Transmission analysis.

Possible patient-to-patient transmission events were determined on the basis of having overlapping stays on the same ward, as well as genetically related *bla*_KPC_ isolates. The analysis was performed separately for two different levels of genetic relatedness (strain or Tn*4401* variant). This is described in detail in the supplemental material.

### Nucleotide sequence accession number.

Sequence data obtained in this study have been deposited at the National Center for Biotechnology Information under BioProject no. PRJNA246471.

## RESULTS

There were 204 patients infected/colonized with *bla*_KPC_-positive Enterobacteriaceae during the prospective sampling period, on the basis of clinical and surveillance sampling. We performed short-read Illumina sequencing of all 294 available isolates; 13 of them were excluded because of quality issues (see Materials and Methods), leaving 281 isolates, from 182/204 (89%) patients, for analysis (see Table S1 in the supplemental material). In all 281 isolates, *bla*_KPC_ was carried within a complete or partial Tn*4401* structure.

### *bla*_KPC_ is found in many different host strains, indicating frequent HGT.

There were 13 different species carrying *bla*_KPC_ (Fig. 1). The four most prevalent species were Enterobacter cloacae (96 isolates from 80 patients), K. pneumoniae (94 isolates from 55 patients), Klebsiella oxytoca (35 isolates from 20 patients), and Citrobacter freundii (30 isolates from 25 patients), each of which showed substantial genetic diversity. Across all of the species, there were a total of 62 distinct strains (>500 chromosomal SNVs; see Materials and Methods). Of these, 18 strains were identified in multiple patients and 44 were seen in only a single patient (Fig. 1), with new strains continuing to appear throughout the study period. The very recent emergence of *bla*_KPC_ on an evolutionary time scale (15) implies that each strain likely acquired *bla*_KPC_ independently, demonstrating frequent HGT between different strains and species.

### The *bla*_KPC_ plasmids pKPC_UVA01 and pKPC_UVA02 are widely dispersed.

We hypothesized that the spread of *bla*_KPC_ could be due to conjugative transfer of the index *bla*_KPC_ plasmids, pKPC_UVA01 and pKPC_UVA02. With plasmid presence defined as ≥99% sequence identity over ≥80% of the plasmid length, 121 (66%) and 32 (18%) patients had isolates carrying pKPC_UVA01 and pKPC_UVA02, respectively, corresponding to 39 and 5 distinct strains from 10 and 4 species, respectively (Fig. 1). Although the wide dispersal of these plasmids supports the plasmid-mediated outbreak hypothesis, short-read data can be limited in providing structural inferences when repetitive sequences are present, and for many isolates, it was not possible to confirm that *bla*_KPC_ was actually colocated within pKPC_UVA01 or pKPC_UVA02 (Fig. 1).

### *bla*_KPC_ is found in many different plasmids, indicating frequent Tn*4401* transposition.

To further investigate *bla*_KPC_ plasmid structures, we performed long-read PacBio sequencing of 17 isolates that were chosen at random from the 281 available, yielding closed *bla*_KPC_ structures in all of the cases. Fifteen isolates had a single *bla*_KPC_ plasmid, and two isolates had two *bla*_KPC_ plasmids, giving a total of 19 *bla*_KPC_ plasmids from the 17 isolates (Table 1). One isolate additionally had a chromosomal insertion of Tn*4401*.

**TABLE 1:** *bla*_KPC_-containing structures ascertained from long-read PacBio sequencing of 17 randomly chosen isolates

Isolate	Species	Patient^*h*^	Date	*bla*_KPC_ plasmid	Size (bp)	Group^*a*^	Within-group genetic change(s)^*b*^	Tn*4401* variant	Flanking sequences^*c*^	Tn*2*-like element^*d*^
CAV1344	*K*. pneumoniae	EP	Dec 2010	pKPC_CAV1344	176,497	Singleton	NA^*i*^	Tn*4401*b-1	GTTCT…GTTCT	Yes
CAV1392	*K*. pneumoniae	EU	Mar 2011	pKPC_CAV1392	43,621	pKPC_UVA01	1 SNV	Tn*4401*b-2	GTTCT…GTTCT	Yes
				NA (chromosomal)	NA	NA	NA	Tn*4401*b-2	AGATA…AGATA	No
CAV1596	*K*. pneumoniae	FK	Apr 2012	pKPC_CAV1596-78	77,801	Singleton	NA	Tn*4401*b-2	GTTCT…GTTCT	Yes
				pKPC_CAV1596-97	96,702	Singleton	NA	Tn*4401*b-2	TATCG…TATCG	No
CAV1099	*K*. oxytoca	AU	Apr 2009	pKPC_CAV1099	113,105	pKPC_UVA02	0 SNVs	Tn*4401*b-1	ATGCA…GGCCA^*e*^	No
CAV1335	*K*. oxytoca	EQ	Dec 2010	pKPC_CAV1335	113,105	pKPC_UVA02	0 SNVs	Tn*4401*b-1	ATGCA…GGCCA^*e*^	No
CAV1374	*K*. oxytoca	ED	Aug 2010	pKPC_CAV1374	332,956	Singleton	NA	Tn*4401*b-1	GTTCT…GTTCT	Yes
CAV1043	*E*. asburiae	L	Mar 2008	pKPC_CAV1043	59,138	Singleton	NA	Tn*4401*b-2	GTTCT…GTTCT	Yes
CAV1176	*E*. cloacae	DN	May 2010	pKPC_CAV1176	90,452	pKPC_CAV1176	0 SNVs	Tn*4401*novel-1	GTTCT…GTTCT	Yes
CAV1311	*E*. cloacae	EO	Jan 2011	pKPC_CAV1311	90,452	pKPC_CAV1176	0 SNVs	Tn*4401*novel-1	GTTCT…GTTCT	Yes
CAV1411	*E*. cloacae	FC	Jun 2011	pKPC_CAV1411	90,452	pKPC_CAV1176	1 SNV, 40-kb inversion	Tn*4401*novel-1	GTTCT…GTTCT	Yes
CAV1669	*E*. cloacae	HV	Aug 2012	pKPC_CAV1669	90,452	pKPC_CAV1176	40-kb inversion	Tn*4401*novel-1	GTTCT…GTTCT	Yes
CAV1668	*E*. cloacae	HQ	Aug 2012	pKPC_CAV1668	43,433	pKPC_UVA01	1 SNV, 188-bp deletion	Tn*4401*novel-1	GTTCT…GTTCT	Yes
CAV1321	*C*. freundii	EG	Nov 2010	pKPC_CAV1321-45	44,846	pKPC_UVA01	1,225-bp insertion	Tn*4401*b-1	GTTCT…GTTCT	Yes
				pKPC_CAV1321-244	243,709	Singleton	NA	Tn*4401*b-1	GTTCT…GTTCT	Yes
CAV1741	*C*. freundii	ER	Oct 2012	pKPC_CAV1741	129,196	pKPC_UVA01	14,960-bp duplication, 70,615-bp insertion	Tn*4401*b-1^*f*^	GTTCT…GTTCT	Yes
CAV1151	*K*. intermedia	CD	Sep 2009	pKPC_CAV1151	43,621	pKPC_UVA01	0 SNVs^*g*^	Tn*4401*b-1	GTTCT…GTTCT	Yes
CAV1320	*E*. aerogenes	EL	Nov 2010	pKPC_CAV1320	13,981	Singleton	NA	Tn*4401*b-1	TTGTT…TTGTT	No
CAV1492	*S. marcescens*	GL	Dec 2011	pKPC_CAV1492	69,158	Singleton	NA	Tn*4401*b-8	TTTTT…TTTTT	No

a Plasmids are defined as belonging to the same group if the sequences are largely identical, allowing for a small number of substitutions and/or rearrangements that may be expected to occur within the outbreak time frame. Different groups have very limited homology outside the Tn*4401* region, indicative of independent integrations into distinct plasmid structures. “Singleton” indicates a plasmid backbone that is distinct from all of the others shown.

b Difference(s) from the reference sequence of that plasmid group, as specified in the previous column.

c Sequences immediately flanking Tn*4401*; generally expected to be identical because of 5-bp target site duplication during transposition (28).

d Tn*4401* integrated into the *tnpA* gene of a Tn*2*-like element.

e No evidence of target site duplication.

f Two copies.

g It is noteworthy that this plasmid from *K*. intermedia CAV1151 is exactly identical to pKPC_UVA01 from *K*. pneumoniae CAV1016, with isolation dates 2 years apart.

h Anonymized patient identifiers are used; they do not represent initials or any other personal information.

i NA, not applicable.

**FIG 2:**
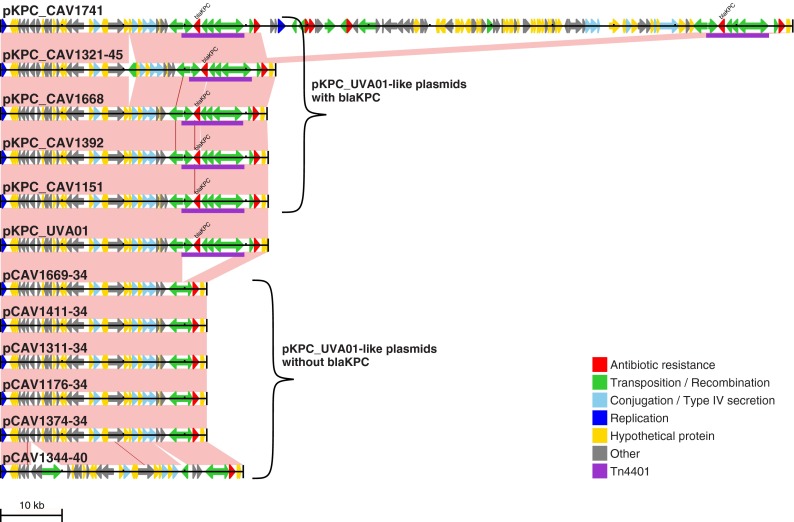
pKPC_UVA01-like plasmids identified through long-read PacBio sequencing. The reference pKPC_UVA01 sequence is shown together with all 11 pKPC_UVA01-like plasmids identified through long-read PacBio sequencing, including the 6 that do not contain *bla*_KPC_. Arrows indicate predicted open reading frames; Tn*4401* is purple. Pink shading indicates regions of identity between adjacent sequences, and SNVs are indicated by red lines.

From the analysis of Illumina data described above, 11 of these 17 isolates contained pKPC_UVA01. As expected, the PacBio assemblies revealed a pKPC_UVA01-like plasmid in each of these isolates. However, only five of these pKPC_UVA01-like plasmids actually contained *bla*_KPC_ (Fig. 2). The other six pKPC_UVA01-like plasmids lacked the entire Tn*4401* element, which was present on a different plasmid in these isolates. Importantly, this demonstrates that plasmid presence (as defined by Illumina sequencing) is an unreliable indicator of the mobile unit carrying *bla*_KPC_, as the “presence” of pKPC_UVA01 was misleading in 55% (6/11) of the randomly selected PacBio isolates.

After accounting for multiple variants of the same plasmid backbone (e.g., the pKPC_UVA01-like plasmids described above), the 19 *bla*_KPC_ plasmids identified through long-read sequencing represented 11 distinct plasmid structures (Table 1; see Fig. S1 in the supplemental material). These consisted of five pKPC_UVA01-like plasmids, two pKPC_UVA02-like plasmids, four pKPC_CAV1176-like plasmids, and eight *bla*_KPC_ plasmids that were each present in only a single PacBio-sequenced isolate. Using Illumina data to assess the presence of each of these 11 distinct *bla*_KPC_ plasmids across the entire set of isolates as described above revealed varied patterns of plasmid presence (Fig. 1). However, in the majority of the cases, it was not possible to determine from Illumina data whether these plasmids contained *bla*_KPC_, so precise details regarding the distribution of *bla*_KPC_-containing plasmids across the 281 isolates remain elusive.

Taken together, these results demonstrate a great deal of *bla*_KPC_ plasmid diversity, as 11 distinct *bla*_KPC_ plasmids were identified through long-read sequencing of 17 isolates. Given that these isolates were randomly chosen, the total number of distinct *bla*_KPC_ plasmids across the entire set of 281 isolates is likely to be much greater than this. Additional Tn*4401* insertion sites were identified from the subset of isolates where flanking sequences could be adequately assembled using short-read data, further supporting this hypothesis (see Table S2 in the supplemental material). Therefore, HGT of the index *bla*_KPC_ plasmids (pKPC_UVA01 and pKPC_UVA02) only partially explains *bla*_KPC_ spread, and the large number of distinct *bla*_KPC_ plasmids indicates high levels of Tn*4401* mobility.

### Tn*4401* is present within a Tn*2*-like element in many different plasmids.

**FIG 3:**
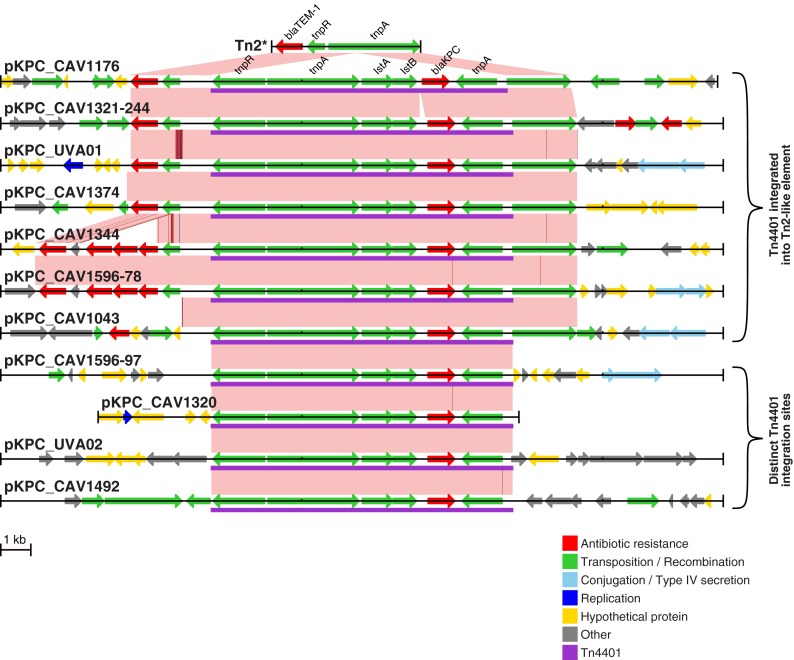
Tn*4401* is commonly integrated into a Tn*2*-like element. Tn*4401* and the surrounding region (i.e., partial plasmid sequence, except for pKPC_CAV1320) are shown for each distinct *bla*_KPC_ plasmid. Variants of the same plasmid backbone (Table 1) are not shown. Arrows indicate predicted open reading frames; Tn*4401* is purple. Pink shading indicates regions of identity between adjacent sequences, SNVs are indicated by red lines, and short indels (1 or 2 bp) are indicated by blue lines. At the very top is the Tn2* reference sequence from AY123253 (49).

In 7 of the 11 distinct, fully characterized *bla*_KPC_ plasmids, Tn*4401* was surrounded by a sequence element related to the *bla*_TEM-1_-containing transposon Tn*2* (Fig. 3). In all of the cases, the Tn*4401* insertion site within the *tnpA* gene of Tn*2* was the same, with approximately 1 kb of flanking sequence on either side of Tn*4401* showing 100% identity, but the remainder of these Tn*2*-like elements showed substantial variation. For example, while the sequence surrounding Tn*4401* in pKPC_CAV1176 was identical to the reference Tn2* sequence, the Tn*2*-like element in pKPC_CAV1043 was truncated. Additionally, pKPC_CAV1344 and pKPC_CAV1596-78 contained a Tn*2* derivative, Tn*1331*, that contains the additional resistance genes *bla*_OXA-9_, *aadA1*, and *aac*(*6*′)-*Ib* and has been seen as a prior Tn*4401* insertion site (46).

### Tn*4401* variation.

There were five different structural variants of Tn*4401* (Table 2). The majority of the isolates, 230/281 (82%), had the Tn*4401*b isoform, with the remaining isolates containing Tn*4401*a (*n* = 8), a novel Tn*4401* isoform with a 188-bp deletion upstream of *bla*_KPC_ (*n* = 39) or one of two truncated Tn*4401* structures (*n* = 4). At the nucleotide level, there were seven sites that were variable within Tn*4401*b. Three of these were located within *bla*_KPC_, giving rise to three different *bla*_KPC_ alleles, *bla*_KPC-2_ (*n* = 179), *bla*_KPC-3_ (*n* = 44), and *bla*_KPC-4_ (*n* = 5). All non-Tn*4401*b isolates contained *bla*_KPC-2_. Taking all structural and nucleotide variations into account, there were a total of 12 different Tn*4401* variants. However, most of these were very rare, with seven found only in single patients.

**TABLE 2:** Tn*4401* variations

Tn*4401* variant^*a*^	Structural isoform (29)	SNV(s)^*b*^	*bla*_KPC_ variant	No. of:		
				Patients	Isolates	Strains
Tn*4401*b-1^*c*^	b		*bla*_KPC-2_	121	176	42
Tn*4401*b-2	b	8015C→T^*d*^	*bla*_KPC-3_	22	40	19
Tn*4401*b-3	b	8015C→T, 9621T→C	*bla*_KPC-3_	1	3	2
Tn*4401*b-4	b	7199T→A, 8015C→T, 9621T→C	*bla*_KPC-3_	1	1	1
Tn*4401*b-5	b	8015N^*e*^	*bla*_KPC-2_/*bla*_KPC-3_	1	2	1
Tn*4401*b-6	b	7509C→G, 7917T→G^*f*^	*bla*_KPC-4_	1	1	1
Tn*4401*b-7	b	6800T→C, 7509C→G, 7917T→G	*bla*_KPC-4_	1	4	1
Tn*4401*b-8	b	9663T→C	*bla*_KPC-2_	1	3	1
Tn*4401*a-1	a (del 7020-7118)		*bla*_KPC-2_	5	8	1
Tn*4401*novel-1	Novel (del 6919-7106)		*bla*_KPC-2_	28	39	2
Tn*4401*trunc-1	Truncated (del 1-6654)		*bla*_KPC-2_	2	3	1
Tn*4401*trunc-2	Truncated (del 1-6727)	6800N^*g*^	*bla*_KPC-2_	1	1	1

a Variants are named such that letters indicate previously described structural isoforms and numbers indicate nucleotide level variations (SNVs) within an isoform (apart from the truncated Tn*4401* structures, where numbers are used to indicate different truncation locations).

b With respect to Tn*4401*b-1, which was considered the reference Tn*4401* sequence in this study.

c Tn*4401*b-1 differs from the reference isoform b sequence in EU176013.1 by the following 14 SNVs: 4939C→G, 4989C→T, 5099A→T, 5131A→G, 5154T→G, 5185G→C, 5255C→A, 5361G→C, 5375C→G, 5390A→C, 5996G→A, 5998G→C, 8112C→A, 8113A→C.

d This substitution converts *bla*_KPC-2_ to *bla*_KPC-3_.

e Quality filters failed at this position because of a mixture of reads supporting C and T (i.e., Tn*4401*b-5 actually represents a mixture of Tn*4401*b-1 and Tn*4401*b-2).

f These two substitutions convert *bla*_KPC-2_ to *bla*_KPC-4_.

g Quality filters failed at this position because of a lack of reads mapped in the reverse direction. All of the reads mapped in the forward direction supported a reference (T) call.

### *bla*_KPC_ mobility has occurred within the hospital.

On the basis of prior health care exposure, the *bla*_KPC_ acquisition source was classified as “imported” (likely acquisition prior to admission to our institution) for 15/182 (8%) patients and “local” (likely acquisition within our institution) for 167/182 (92%) patients (Fig. 1; see Materials and Methods). Imports were more likely to be infected/colonized with K. pneumoniae, particularly ST258 (see Table S3 in the supplemental material), consistent with previous reports of this strain being the dominant *bla*_KPC_ carrier in the United States (9, 47). Thus, most host strain variation likely originated within the hospital via *bla*_KPC_ HGT. In support of this, 15/16 (94%) patients infected/colonized with multiple strains/species had shared Tn*4401* variants within the patient (see Table S4 in the supplemental material), suggesting recent *bla*_KPC_ HGT. Notably, this included one patient with two different species carrying Tn*4401*b-3, which is not found in any other patient.

There was also some evidence of recent within-strain Tn*4401* transposition. From the isolates that were randomly chosen for long-read sequencing, 4/17 (24%) had multiple Tn*4401* copies (Table 1). If we assume that this randomly chosen subset is representative, this extrapolates to approximately 66/281 isolates across the whole data set. However, only 2/281 isolates had multiple Tn*4401* variants (Tn*4401*b-5; Table 2), indicating that many isolates likely had multiple copies of the same Tn*4401* variant, consistent with recent Tn*4401* transposition.

Taken together, these results indicate that much of the genetic diversity observed is due to recent *bla*_KPC_ mobility, likely within the hospital ecosystem over the described 5-year outbreak.

### Direct patient-to-patient transmission does not explain *bla*_KPC_ acquisition.

**FIG 4:**
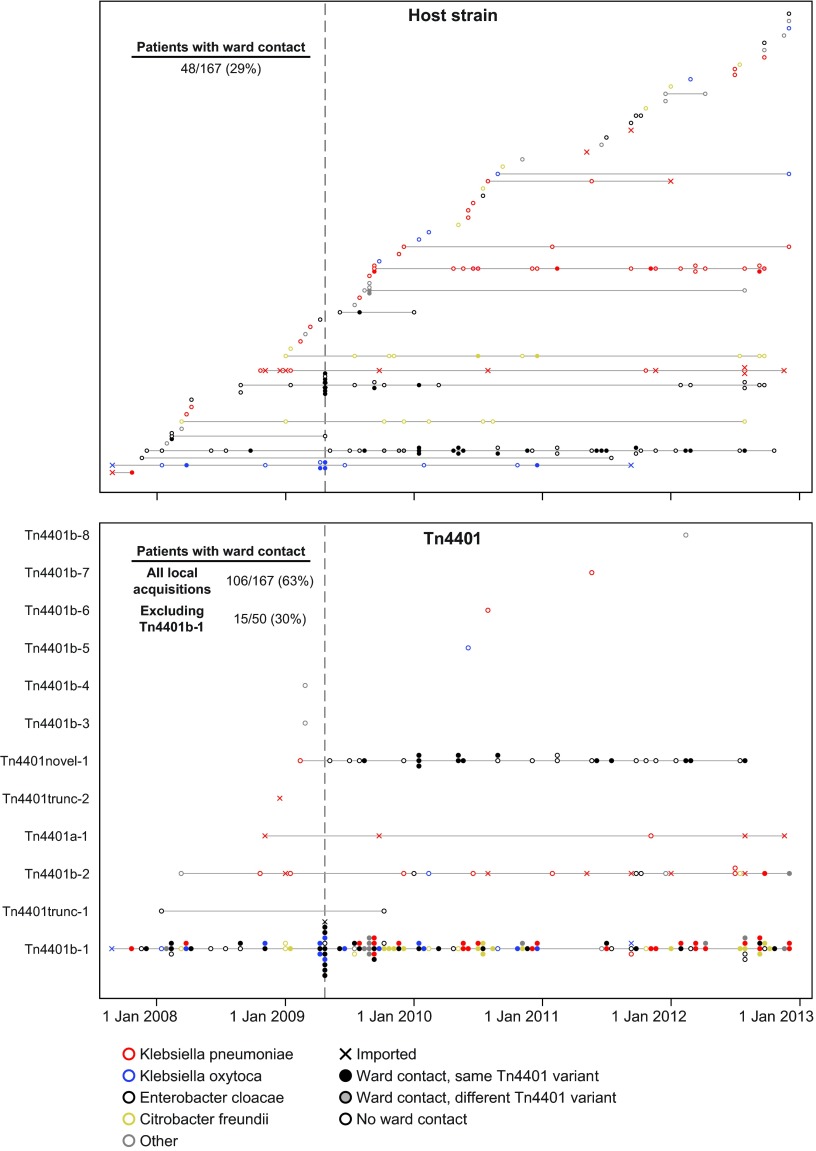
Ward contacts between patients with genetically related isolates. Each horizontal line represents a different strain (top) or Tn*4401* variant (bottom). Filled circles indicate patients who had previous ward contact with another patient on the same horizontal line (i.e., possible patient-to-patient transmission). As Tn*4401*b-1 is present in two-thirds of the patients, many coincidental ward contacts may be expected to occur, resulting in a substantial overestimate of the number of transmission events. Therefore, the total number of Tn*4401* acquisitions explainable by direct ward contact is indicated, as well as that with Tn*4401*b-1-carrying patients excluded. The vertical line indicates the onset of routine patient screening.

To further investigate the *bla*_KPC_ acquisition source, we combined epidemiological and genetic data to trace possible transmission chains at two different genetic levels. We considered possible transmission events where the donor and recipient were on the same ward at the same time and carried the same host strain or Tn*4401* variant. Considering only “local” acquisitions (see above), 48/167 (29%) patients had ward contact with another patient carrying the same *bla*_KPC_-positive strain (Fig. 4, top). A greater proportion of the patients, 106/167 (63%), had ward contact with another patient carrying the same Tn*4401* variant. However, as Tn*4401*b-1 is very common (66% of the patients), these inferred transmissions may be spurious. With patients carrying this common variant excluded, only 15/50 (30%) had ward contact with another patient carrying the same Tn*4401* variant (Fig. 4, bottom). Therefore, both genetic levels (strain or Tn*4401* variant) demonstrated plausible transmissions for only a minority of the patients, indicating that direct patient-to-patient transmission is not the dominant mode of *bla*_KPC_ acquisition or that there are many silently colonized patients below the limit of detection by our surveillance methods (40, 48).

## DISCUSSION

Here we have demonstrated high levels of genetic diversity in KPC-producing Enterobacteriaceae within a single institution over 5 years. This diversity occurs at multiple genetic levels, revealing a complex evolutionary history of the *bla*_KPC_ gene involving many different host strains and plasmids.

In 7/11 distinct *bla*_KPC_ plasmids identified through long-read sequencing, Tn*4401* was located within a Tn*2*-like element. As these Tn*2*-like elements differed substantially from each other (Fig. 3), it is unlikely that this arose via the transposition of a composite Tn*4401*-Tn*2*-like structure. Instead, it suggests that Tn*4401* has been repeatedly incorporated into pre-existing Tn*2*-like elements, which are known to be widespread, and genetically divergent, in Enterobacteriaceae (49, 50). However, the insertion site was identical in all of the cases, yet Tn*4401* has been reported to have no insertion site specificity (28), suggesting that this was not facilitated by a standard transposition mechanism. Therefore, we suggest that this is most likely mediated by homologous recombination with other Tn*2*-like elements following an initial integration event, as recently suggested for another multidrug resistance gene, *bla*_CTX-M-15_ (51). This implies that Tn*4401* mobility may have been enhanced via integration into a second, already widely dispersed, transposon. As the Tn*4401*-Tn*2*-like structure was present in the index case isolate (CAV1016, August 2007), we presume that the initial transposition of Tn*4401* into a Tn*2*-like element occurred prior to entry into our hospital system. In support of this, one particular Tn*2*-like element, Tn*1331*, has been previously reported to contain Tn*4401* (in exactly the same position within the *tnpA* gene as that described here) (21, 46, 52, 53), including one report describing a K. pneumoniae isolated in 2005, which predates *bla*_KPC_ in our institution (46). We are not aware of any previous reports describing Tn*4401* within a non-Tn*1331* Tn*2*-like element.

The prevalence of Tn*4401* insertions within Tn*2*-like elements also has important implications with regard to plasmid tracking. We previously published a method for arbitrary PCR to track the flanking regions around the Tn*4401* element, as well as a PCR method to assay the presence of what we had wrongly assumed was a single plasmid, pKPC_UVA01. This PCR assay targeted the immediate Tn*4401* insertion site within a Tn*2*-like element (54), which we have demonstrated here is present in many different plasmids, highlighting that PCR assay results, and indeed, those of any partial typing methods, need to be interpreted with a great deal of caution. We were further misled by the analysis of short-read whole-genome sequencing data that indicated the presence of pKPC_UVA01 in the majority of our isolates. Taking these findings together, it was tempting to conclude that horizontal transfer of pKPC_UVA01 was responsible for the great majority of the *bla*_KPC_ carriage at our institution. However, long-read sequencing refuted this, revealing a far more complex picture.

More generally, this highlights certain limitations of plasmid reconstruction from short-read data. To illustrate by way of example, there were five isolates where long-read sequencing revealed pKPC_UVA01-like plasmids that were identical to the reference pKPC_UVA01 sequence apart from the absence of Tn*4401* and the associated 5-bp target site duplication (Fig. 2). We presume that in these lineages, *bla*_KPC_ may have been initially acquired via HGT of pKPC_UVA01, with subsequent homologous recombination transferring Tn*4401* from pKPC_UVA01 to a different plasmid containing a Tn*2*-like element. In each of these five isolates, there are multiple Tn*2*-like elements that have 100% sequence identity over approximately 1 kb on either side of the Tn*4401* insertion site. As this is longer than the fragment length used for paired-end sequencing, it is not possible to resolve the plasmid context of *bla*_KPC_ by using short-read data. Importantly, any reference-based method for plasmid reconstruction (e.g., in this case, using the pKPC_UVA01 reference sequence to infer the presence of the plasmid in each isolate) is liable to produce misleading results. More generally, it is exactly the repetitive regions that cannot be resolved by using short-read data that could be expected to be involved in plasmid rearrangements, either through homologous recombination, as suggested here, or by virtue of the fact that transposable elements are often present in multiple copies. Therefore, having short-read data that are consistent with a known plasmid structure, even within the same outbreak, should not be sufficient to conclude that that structure is present, if the data are also consistent with an alternative structure. As several recent studies have utilized reference-based approaches for plasmid assembly/inference (33, 34), our results indicate that results obtained by any such methods should be interpreted with extreme caution.

Across the *bla*_KPC_-positive patients, there was large variation in both host strains and *bla*_KPC_ plasmids, with Tn*4401* being the largest genetic unit that was consistently present. Therefore, surveillance strategies aimed at tracking individual strains or plasmids could be misleading, and it may be more appropriate to focus on Tn*4401*. However, we found limited variation within the transposon, as Tn*4401* sequences from 121/182 (66%) patients were identical to the index case (Table 2). This lack of variation implies that even the highest-resolution genetic methods may be insufficient for determining specific transmission routes. Even so, we have demonstrated that only a minority of *bla*_KPC_ acquisition events can be explained by direct patient-to-patient transmission. Future studies should therefore contemporaneously investigate the possible involvement of unsampled reservoirs (e.g., environmental or silent colonization by additional carriers).

There are several limitations to this study. Because of the cost and effort involved in long-read sequencing, we were able to resolve only a minority of *bla*_KPC_ plasmids. This means that although we have a compelling indicator of the diversity created by mobile genetic elements within a single hospital over a 5-year period, we are limited in the ability to genetically resolve pathways of *bla*_KPC_ mobility between host strains and plasmid vectors, even within a single patient. We also speculate about the effect of Tn*4401* insertion into Tn*2*-like elements, but future *in vitro* studies could be used to illuminate the effect of this composite structure on Tn*4401* mobility. Another issue is the limit of detection of the culture-based screening methods and phenotypic tests used to identify *bla*_KPC_-positive clinical isolates. No single perirectal screening method to capture asymptomatically colonized patients is perfect (40, 48, 55), including the method used here, which has a sensitivity of ∼86% (40). In recognition of the fact that *bla*_KPC_ expression and carbapenem susceptibility may be variable in different host species, and in the context of additional resistance mechanisms such as porin alterations, we lowered our surveillance thresholds to include all of the possible ESBL-producing organisms that subsequently tested positive in phenotypic carbapenemase tests. Even with this mitigation strategy, we anticipate that we have missed a proportion of the *bla*_KPC_-positive Enterobacteriaceae isolates that may be contributing to the evolution and transmission of *bla*_KPC_ within our institution. Overall however, our broad screening approach across the members of the family Enterobacteriaceae has highlighted the importance of species other than E. coli and Klebsiella spp. in the transmission of *bla*_KPC_, with implications for the current CDC rectal surveillance protocol.

**FIG 5:**
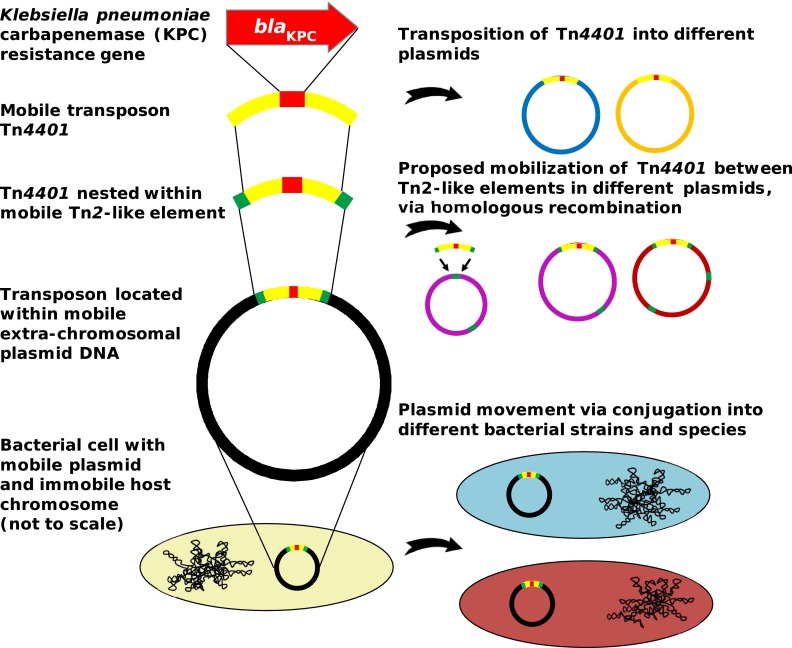
*bla*_KPC_ spreads at multiple genetic levels, resulting in a high level of diversity in *bla*_KPC_-positive Enterobacteriaceae isolates.

In conclusion, our detailed genetic analysis of the evolutionary events occurring in the early stages of antimicrobial resistance gene emergence in a single institution identifies several distinct processes occurring at high frequency (Fig. 5). First, the presence of shared *bla*_KPC_-containing strains in different patients reflects traditional (clonal) outbreak models. Second, *bla*_KPC_ mobility between strains/species is facilitated by promiscuous *bla*_KPC_ plasmids such as pKPC_UVA01. Third, *bla*_KPC_ transfer between plasmids is likely enhanced by homologous recombination between Tn*2*-like elements, facilitating the movement of Tn*4401* from one plasmid to another. Finally, *bla*_KPC_ mobility is also enabled by standard Tn*4401* transposition. Rather than a single process dominating, resistance dissemination is driven by a combination of these factors, with mobility occurring at multiple nested genetic levels, analogous to a Russian doll set. This has resulted in a high level of diversity in KPC-producing Enterobacteriaceae, at multiple genetic levels. As *bla*_KPC_ prevalence continues to increase, so will this genetic diversity, inevitably resulting in a wider variety of more pathogenic strains carrying *bla*_KPC_.

Our results indicate that the current standard practice of screening only specific species for *bla*_KPC_ carriage is likely to hamper surveillance efforts by grossly underestimating its true prevalence. Instead of the traditional view of an outbreak involving a single pathogenic strain, we propose that for KPC-producing Enterobacteriaceae, and possibly more generally, we should instead adopt the view of a “gene-based outbreak,” with surveillance strategies tracking the resistance gene itself rather than a specific host strain.

## Supplementary Material

Supplemental material
