# Supplementary material for: Nested Russian Doll-Like Genetic Mobility Drives Rapid Dissemination of the Carbapenem Resistance Gene blaKPC
Source: Antimicrob Agents Chemother. 2016 May 23;60(6):3767–78. doi: 10.1128/AAC.00464-16 (PMC4879409; doi:10.1128/AAC.00464-16)
Supplement: Supplemental material [file supp_60_6_3767__index.html]

Nested Russian Doll-Like Genetic Mobility Drives Rapid Dissemination of the Carbapenem Resistance Gene blaKPC — Supplemental material 

# Nested Russian Doll-Like Genetic Mobility Drives Rapid Dissemination of the Carbapenem Resistance Gene *bla*KPC

## Supplemental material

- Supplemental file 1 -

  Supplemental Methods, Tables S2-S5, Figure S1

  PDF, 828K
- Supplemental file 2 -

  Table S1

  XLSX, 30K
