# Supplementary material for: Nested Russian Doll-Like Genetic Mobility Drives Rapid Dissemination of the Carbapenem Resistance Gene blaKPC
Source: Antimicrob Agents Chemother. 2016 May 23;60(6):3767–78. doi: 10.1128/AAC.00464-16 (PMC4879409; doi:10.1128/AAC.00464-16)
Supplement: Supplemental material [file AAC.00464-16_zac006165250so1.pdf]

Supplementary Methods

Isolate collection and Illumina sequencing

Illumina sequencing was performed as previously (1), with the exception of three isolates, CAV1602, CAV1606 and CAV1697. For these, genomic DNA was extracted using a QIAcube automated sample preparation station (Qiagen, San Diego, CA) and libraries for sequencing were generated and normalized using the Nextera XT DNA library preparation kit (Illumina, San Diego, CA). Whole genome sequencing was performed on a MiSeq benchtop sequencer (Illumina, San Diego, CA, USA), utilizing 500 cycles of paired-end reads.

Isolates from 204 patients were prospectively collected and identified as having bla_{KPC} by PCR, however a small number of these isolates were not viable, were incorrectly labelled, lost bla_{KPC} through laboratory passage or were contaminated during the cataloguing process. We initially performed Illumina sequencing on 294 available bla_{KPC}-positive isolates. In ten isolates, the entire Tn*4401* region (including bla_{KPC}) was absent from the sequencing data (as determined by BLASTn comparisons with the isolate's *de novo* assembly), presumably due to plasmid loss in culture. Three additional isolates showed inconsistencies in species classification (see below). These 13 isolates were therefore excluded, leaving 281 isolates from 182 patients for analysis.

Species classification

Species classification was initially performed in the clinical microbiology lab using the VITEK2 system with the GN ID card (bioMérieux, Durham, NC). To provide an independent classification method, Illumina sequenced reads were taxonomically assigned using Kraken version 0.10.4-beta (2) with the MiniKraken database from March 30th, 2014. The top species was considered as a positive match if >20% of the reads were assigned to this species. If <10% of reads were assigned to the top species, we postulated that the correct species was most likely not in the MiniKraken database. In this case, a presumed species was determined from the microbiological classification and/or similarity in the Kraken results to other isolates with more definitive microbiological classifications (this applied to *Citrobacter amalonaticus*, *Citrobacter freundii*, and *Kluyvera intermedia*). All isolates were then mapped to species-specific references, where available, and species classification was confirmed by the quality of this mapping. For three related isolates from patient AM, the microbiology lab was able to classify only to family level (*Enterobacteriaceae*), and a species could not be determined by the sequence-based method; these are considered to represent an unknown species and have been labelled as 'Other'. Three further isolates produced inconsistent results from the two classification methods (possible labelling errors) and were therefore excluded from further analyses.

Phylogenetic analysis and strain classification

For each species represented by at least three isolates after the removal of within-patient duplicates (see main Methods), phylogenetic analysis was performed using PhyML version 20120412 (3) with an alignment consisting of all variable sites as identified through mapping to a species-specific chromosomal reference (Supplementary Table 5), padded to the length of the reference with invariant sites of the same GC content as the original data. For species

represented by only two isolates, PhyML could not be used. In these cases tree topology is unambiguous and branch length was taken to be the proportion of variant sites between the two isolates.

Genetic clusters were defined using the above phylogenies as follows: Isolates were considered to belong to the same strain if they both descended (directly or indirectly) from an internal node with at least one path to a tip consisting only of branches with length $<10^{-4}$ (corresponds to 500 SNVs for a 5 Mb chromosome). This essentially partitions the tree on long branches, but as the cutoff used for a long branch is deliberately quite high, the method will tend to overcluster (i.e. it is conservative in the definition of a chromosomally distinct strain).

Long-read PacBio sequencing

To refine each of the initial PacBio assemblies, Illumina reads from the corresponding isolate were mapped using bwa-mem version 0.7.5a-r405 (4) and visualised using Gap5 v1.2.14-r (5). Unmapped reads were *de novo* assembled using a5-miseq version 20140401 (6) to identify small plasmids, as these were expected to be absent from the PacBio assembly due to size selection of the input DNA (i.e. <7kb DNA fragments were removed). Plasmid and chromosome structures were closed by resolving repeats at the ends of contigs. As the repeats were generally imperfect, mapping information was used to determine the correct sequence in each case. To validate the final assembly structures, each replicon was re-linearised in a non-repeat region, the Illumina reads were remapped, and Gap5 was used for visualisation, confirming the absence of misassemblies. The consensus sequence from mapping was also used to correct minor errors in the assembly sequence (generally single base indels in homopolymeric regions.

For one isolate (CAV1344), closed bla_{KPC} plasmid structure(s) could not be obtained and further investigation revealed the most likely cause to be a mixture of plasmid structures in the DNA population that was sequenced. Therefore, we re-isolated DNA from a single colony and repeated PacBio sequencing, which was then successful. We report the results of the latter sequencing, although it should be noted that our Illumina sequence data was obtained from the presumably mixed population.

Analysis of Tn*4401* flanking sequences

For each plasmid that was classified as being present, the *de novo* assembly was further examined to determine, if possible, whether Tn*4401* was in fact contained within this plasmid in the expected sequence context. For plasmids where Tn*4401* was not inserted into a Tn*2*-like element, 400 bp of flanking sequence from each side of Tn*4401* in the reference plasmid sequence was used to query the *de novo* assembly using BLASTn. For each side, if there was a single contig covering the entire 400 bp region, 400 bp of sequence adjacent to the match was compared with the expected Tn*4401* sequence, as well as the sequence on the other side of Tn*4401* in the plasmid reference. If both sides matched the expected Tn*4401* sequence, then the plasmid was classified as containing Tn*4401* in that isolate. If the two sides matched each other over a length of ≥300 bp (i.e. the insertion site was assembled without containing Tn*4401*, but possibly with minor indels) then the plasmid was classified as not containing Tn*4401*. In any other situation (generally if there were contig breaks at or close to the Tn*4401* insertion sites) then the plasmid was classified as uncertain.

For plasmids where Tn*4401* was inserted into a Tn*2*-like element, the immediate sequence flanking Tn*4401* would not be sufficient to distinguish between these different plasmids. Therefore, to classify one of these plasmids as containing Tn*4401*, we required that for each side of Tn*4401*, the entire Tn*2* region from the reference plasmid, along with 400 bp on either side (i.e. 400 bp of Tn*4401* sequence, the Tn*2* region, and 400 bp of sequence specific to that plasmid) was present in the *de novo* assembly as a continuous sequence on a single contig. To classify one of these plasmids as not containing Tn*4401*, the same method was used as for non-Tn*2* plasmids above. In other words, if the region of Tn*2* encompassing the Tn*4401* integration site was assembled as a continuous sequence on a single contig, then we assumed that Tn*4401* could not be integrated into a Tn*2*-like element in any plasmid in that isolate.

For the identification of novel Tn*4401* insertion sites without a flanking Tn*2*-like element, a similar method was used, with the initial query sequence consisting of 400 bp at either end of Tn*4401*. The adjacent sequences were then compared with the bla_{KPC} plasmids identified from long-read sequencing, and we report isolates where at least 400 bp of flanking sequence on either side of Tn*4401* could be determined, and had little/no homology to any of the known PacBio bla_{KPC} plasmids.

Epidemiological classification

For each patient, bla_{KPC} acquisition source was epidemiologically classified as either "local" (likely acquisition within UVaMC) or "imported" (likely acquisition prior to UVaMC admission), on the basis of hospitalisation time at UVaMC prior to bla_{KPC} isolation, with an arbitrary 48h cutoff (see main Methods for details). While this may have resulted in a small number of misclassifications, this is expected to be minimal, for the following reasons.

Firstly, extensive surveillance screening was performed: Beginning April 27th 2009, weekly perirectal screening was performed for all patients from selected high risk patient care units where a persistently high incidence of carbapenamase-producing *Enterobacteriaceae* (CPE) was detected, as well as inpatient units caring for a patient known to be colonized or infected with CPE (for example this resulted in 6860 surveillance cultures performed in 2012) (7, 8). Additionally, the median length of hospital stay immediately prior to first bla_{KPC}-positive isolate for the 167 local acquisitions with a sequenced isolate was 13 days (IQR 4-28.5 days), with 62/130 (48%) local acquisitions following screening onset having at least one negative surveillance culture prior to the first positive, 45/62 (73%) of which were within the same hospital stay. Given the extent of screening, the length of hospitalisation prior to bla_{KPC} isolation, and the presence of prior negative cultures in many cases, the classification of "local" acquisitions should be robust.

With respect to the robustness of the "imported" classification, all but one of the imports had recent outside hospital exposure, consistent with bla_{KPC} *Enterobacteriaceae* being healthcare acquired. The one exception had a bla_{KPC} isolate with 0 SNV differences relative to two other isolates from other patients in the same ward on the same day, indicating that in this case bla_{KPC} was likely acquired within the first 48h of admission (i.e. a false "imported" classification).

Transmission analysis

An upper bound for the number of potential patient-to-patient transmission events was determined by identifying all possible donor-recipient pairs from the 182 patients with sequenced isolates where the donor and recipient patients were on the same ward at the same time at least one day prior to the first isolation of a bla_{KPC}-positive *Enterobacteriaceae* isolate from the recipient. Patients were considered at risk for being a donor of bla_{KPC}-positive *Enterobacteriaceae* at any point during hospitalization (i.e. before as well as after their first positive isolate, conservatively including all time in hospital even if the patient had previous negative cultures), and were considered to be carriers for the duration of the study. Potential donor-recipient pairs with shared bacterial strains (<200 SNVs) or Tn*4401* variants were then identified. For each level of genetic relatedness (strain or Tn*4401* variant), a transmission network was constructed and plausible transmission events were identified to maximise the number of potential recipients using a method adapted from (9).

References

1. **Mathers AJ, Stoesser N, Sheppard AE, Pankhurst L, Giess A, Yeh AJ, Didelot X, Turner SD, Sebra R, Kasarskis A, Peto T, Crook D, Sifri CD.** 2015. Klebsiella pneumoniae carbapenemase (KPC) producing K. pneumoniae at a Single Institution: Insights into Endemicity from Whole Genome Sequencing. Antimicrob Agents Chemother.
2. **Wood DE, Salzberg SL.** 2014. Kraken: ultrafast metagenomic sequence classification using exact alignments. Genome Biol **15:**R46.
3. **Guindon S, Gascuel O.** 2003. A simple, fast, and accurate algorithm to estimate large phylogenies by maximum likelihood. Syst Biol **52:**696-704.
4. **Li H.** 2013. Aligning sequence reads, clone sequences and assembly contigs with BWA-MEM.arXiv:1303.3997v1301 [q-bio.GN].
5. **Bonfield JK, Whitwham A.** 2010. Gap5--editing the billion fragment sequence assembly. Bioinformatics **26:**1699-1703.
6. **Coil D, Jospin G, Darling AE.** 2015. A5-miseq: an updated pipeline to assemble microbial genomes from Illumina MiSeq data. Bioinformatics **31:**587-589.
7. **Enfield KB, Huq NN, Gosseling MF, Low DJ, Hazen KC, Toney DM, Slitt G, Zapata HJ, Cox HL, Lewis JD, Kundzins JR, Mathers AJ, Sifri CD.** 2014. Control of simultaneous outbreaks of carbapenemase-producing enterobacteriaceae and extensively drug-resistant Acinetobacter baumannii infection in an intensive care unit using interventions promoted in the Centers for Disease Control and Prevention 2012 carbapenemase-resistant Enterobacteriaceae Toolkit. Infect Control Hosp Epidemiol **35:**810-817.
8. **Mathers AJ, Poulter M, Dirks D, Carroll J, Sifri CD, Hazen KC.** 2014. Clinical Microbiology Costs for Methods of Active Surveillance for Klebsiella pneumoniae Carbapenemase-Producing Enterobacteriaceae. Infect Control Hosp Epidemiol **35:**350-355.
9. **Walker AS, Eyre DW, Wyllie DH, Dingle KE, Harding RM, O'Connor L, Griffiths D, Vaughan A, Finney J, Wilcox MH, Crook DW, Peto TE.** 2012. Characterisation of Clostridium difficile hospital ward-based transmission using extensive epidemiological data and molecular typing. PLoS Med **9:**e1001172.

Table S1. Details of sequenced isolates. (Provided as a separate file)

Table S2. Additional Tn*4401* insertion sites ascertained from short-read Illumina data

Patients	Isolates	Species	Genetic cluster	Length left (kb)[a]	Length right (kb)[a]	Tn*4401* variant	Flanking sequence[b]
FA	CAV1417, CAV1427, CAV1471, CAV1550	*K. pneumoniae*	Kpne-4	5	28	Tn*4401*b-7	ATGAA…ATGAA
AD	CAV1062, CAV1127	*K. pneumoniae*	Kpne-2	4	3	Tn*4401*a-1	ATTGA…GGTTT[c]
FY	CAV1453, CAV1477	*K. pneumoniae*	Kpne-2	111	136	Tn*4401*a-1	AATAA…CTATT[c]
CI, IV	CAV1378, CAV1746	*K. pneumoniae*	Kpne-2	2	7	Tn*4401*a-1	ATTGA…ATTGA
AM	CAV1074	*K. pneumoniae*	NA	2	1	Tn*4401*b-3	AATAA…AATAA
IN	CAV1752	*K. oxytoca*	NA	32	52	Tn*4401*b-1	AACAA…AACAA
FR	CAV1459	*E. cloacae*	Eclo-5	3	2	Tn*4401*b-2	GTTTT…GTTTT

[a] Length of flanking sequence on the same contig as each end of Tn*4401*
[b] Sequences immediately flanking Tn*4401*; generally expected to be identical due to 5 bp target site duplication during transposition
[c] No evidence of target site duplication

Table S3. Association of importation status with *K. pneumoniae* and the epidemic *bla*$_{KPC}$ *K. pneumoniae* strain ST258

Source of acquisition	*K. pneumoniae*[a]	No *K. pneumoniae*[b]	*K. pneumoniae* ST258[c]	*K. pneumoniae*, no ST258[d]
Imported	12	3	9	3
Local	43	124	3	40
	p<0.0001[e]		p<0.0001[e]	

[a] Patients with at least one *K. pneumoniae* isolate (includes patients with *K. pneumoniae* and another species)
[b] Patients with no *K. pneumoniae* isolates (excludes patients with *K. pneumoniae* and another species)
[c] Patients with at least one *K. pneumoniae* ST258 isolate (includes patient FK who has both ST258 *K. pneumoniae* and non-ST258 *K. pneumoniae*)
[d] Patients with at least one *K. pneumoniae* isolate, but no ST258 (excludes patient FK)
[e] Fisher's exact test

Table S4. Patients with multiple *bla*$_{KPC}$-positive strains or species

Patient	Species	Strain	Tn*4401* variant
B	*K. pneumoniae*	Kpne-6	Tn*4401*b-1
	K. oxytoca	Koxy-1	Tn*4401*b-1
AF	*C. freundii*	Cfre-2	Tn*4401*b-1
	E. cloacae	Eclo-2	Tn*4401*b-1
CB	*R. ornothinolytica*	NA	Tn*4401*b-1
	K. pneumoniae	NA	Tn*4401*b-1
DJ	*E. cloacae*	Eclo-2	Tn*4401*b-1
	K. pneumoniae	NA	Tn*4401*b-1
DU	*K. pneumoniae*	Kpne-1	Tn*4401*b-1
	E. cloacae	NA	Tn*4401*b-1
ED	*K. oxytoca*	Koxy-2	Tn*4401*b-1
	C. freundii	NA	Tn*4401*b-1
GV	*E. cloacae*	Eclo-2	Tn*4401*b-1
	K. oxytoca	NA	Tn*4401*b-1
HA	*E. aerogenes*	NA	Tn*4401*b-1
	K. oxytoca	Koxy-2	Tn*4401*b-1
IN	*K. pneumoniae*	Kpne-1	Tn*4401*b-1
	K. oxytoca	NA	Tn*4401*b-1
AK	*K. pneumoniae*	NA	Tn*4401*novel-1
	E. cloacae	Eclo-1	Tn*4401*novel-1
L	*E. asburiae*	NA	Tn*4401*b-2
	K. pneumoniae	NA	Tn*4401*b-2
FK	*K. pneumoniae*	NA	Tn*4401*b-2
	K. pneumoniae	Kpne-2	Tn*4401*b-2
IL	*K. pneumoniae*	NA	Tn*4401*b-2
	C. amalonaticus	NA	Tn*4401*b-2
	E. cloacae	NA	Tn*4401*b-2
JT	*E. aerogenes*	NA	Tn*4401*b-2
	E. cloacae	NA	Tn*4401*b-2
AM	Unknown	NA	Tn*4401*b-3/4[a]
	K. pneumoniae	NA	Tn*4401*b-3
GL	*S. marcescens*	Smar-1	Tn*4401*b-8
	C. freundii	Cfre-2	Tn*4401*b-1

[a] Two isolates with Tn*4401*b-3 and one isolate with Tn*4401*b-4. Note that both Tn*4401*b-3 and Tn*4401*b-4 are exclusive to patient AM

Table S5. Chromosomal references used for mapping

Species	Number of Isolates	Reference strain	Reference Length (bp)	Median % reference sites called (range)	Accession number
Citrobacter amalonaticus	2	CAV1321 (*Citrobacter freundii*)[a]	4,976,908	60 (60-61)	CP011612
Citrobacter freundii	30	CAV1321[b]	4,976,908	95 (81-98)	CP011612
Enterobacter aerogenes	4	EA1509E	5,419,609	88 (87-89)	NC_020181.1
Enterobacter asburiae	1	NCTC 9394 (*Enterobacter cloacae*)[c]	4,908,759	70 (70-70)	NC_021046.1
Enterobacter cloacae	96	NCTC 9394	4,908,759	85 (71-89)	NC_021046.1
Escherichia coli	2	DH10B	4,686,137	80 (78-83)	NC_010473.1
Klebsiella pneumoniae	94	MGH78578	5,315,120	88 (82-90)	CP000647.1
Klebsiella oxytoca	35	E718	6,097,032	79 (76-91)	NC_018106.1
Kluyvera intermedia	7	CAV1151[b]	5,529,132	97 (96-97)	CP011602
Proteus mirabilis	1	HI4320	4,063,606	87 (87-87)	NC_010554.1
Raoultella ornothinolytica	1	B6	5,398,151	91 (91-91)	NC_021066.1
Serratia marcescens	5	WW4	5,241,455	81 (80-81)	NC_020211.1
Other (unknown)	3	NCTC 9394 (*Enterobacter cloacae*)[a]	4,908,759	46 (46-46)	NC_021046.1

[a] No species-specific reference available

[b] Comparisons made with long-read (PacBio), fully closed chromosomes for these strains obtained in this study

[c] *E. cloacae* reference used as *E. asburiae* is part of the *E. cloacae* complex

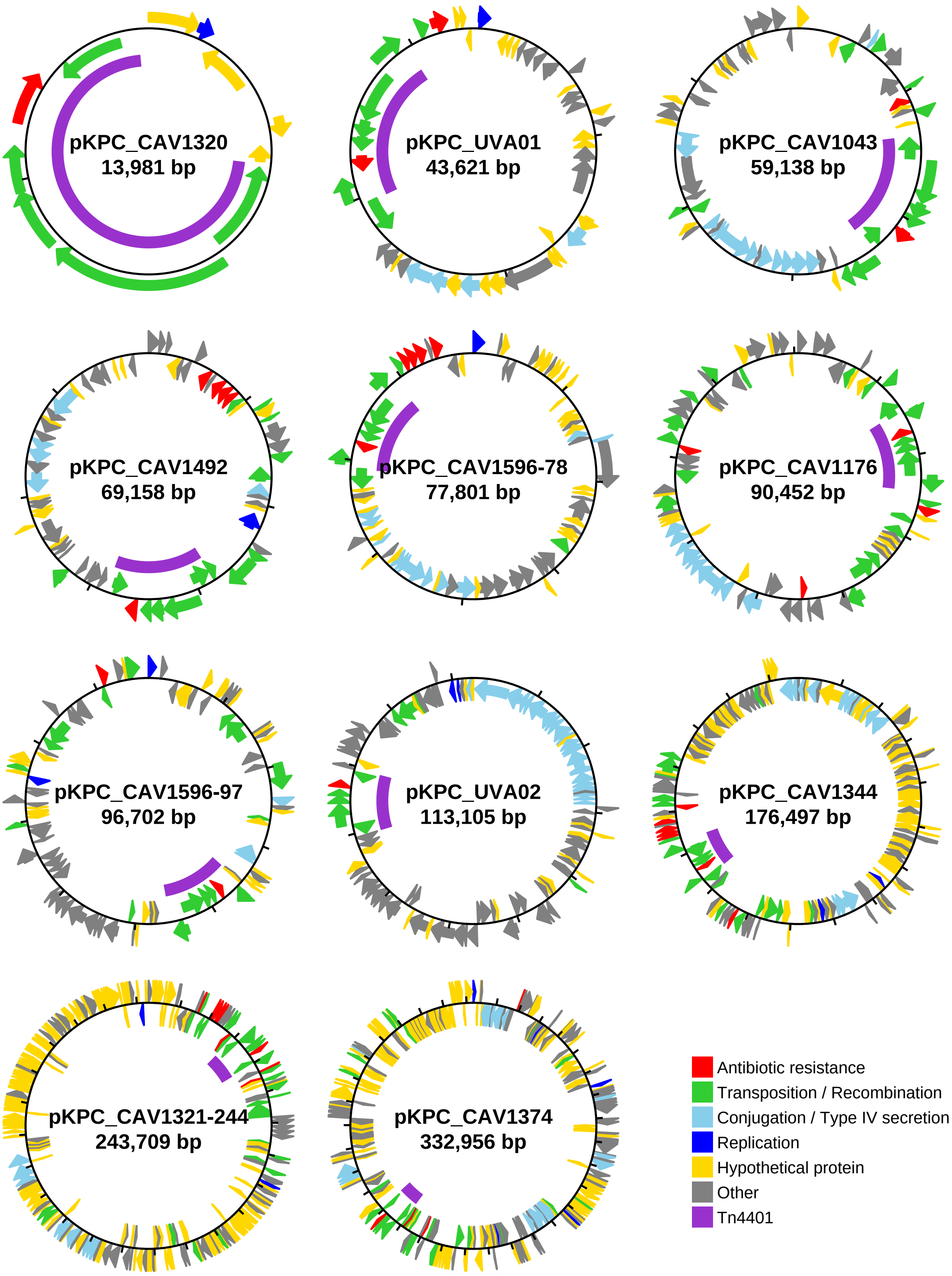

Figure S1. Distinct *bla*$_{KPC}$ plasmids identified through long-read PacBio sequencing. Variants of the same plasmid backbone (see Table 1) are not shown. Arrows indicate predicted open reading frames; Tn*4401* is shown in purple.
